# Blind Video Quality Assessment for Ultra-High-Definition Video Based on Super-Resolution and Deep Reinforcement Learning

**DOI:** 10.3390/s23031511

**Published:** 2023-01-29

**Authors:** Zefeng Ying, Da Pan, Ping Shi

**Affiliations:** School of Information and Communication Engineering, Communication University of China, Beijing 100024, China

**Keywords:** blind video quality assessment, deep reinforcement learning, ultra-high-definition video

## Abstract

Ultra-high-definition (UHD) video has brought new challenges to objective video quality assessment (VQA) due to its high resolution and high frame rate. Most existing VQA methods are designed for non-UHD videos—when they are employed to deal with UHD videos, the processing speed will be slow and the global spatial features cannot be fully extracted. In addition, these VQA methods usually segment the video into multiple segments, predict the quality score of each segment, and then average the quality score of each segment to obtain the quality score of the whole video. This breaks the temporal correlation of the video sequences and is inconsistent with the characteristics of human visual perception. In this paper, we present a no-reference VQA method, aiming to effectively and efficiently predict quality scores for UHD videos. First, we construct a spatial distortion feature network based on a super-resolution model (SR-SDFNet), which can quickly extract the global spatial distortion features of UHD videos. Then, to aggregate the spatial distortion features of each UHD frame, we propose a time fusion network based on a reinforcement learning model (RL-TFNet), in which the actor network continuously combines multiple frame features extracted by SR-SDFNet and outputs an action to adjust the current quality score to approximate the subjective score, and the critic network outputs action values to optimize the quality perception of the actor network. Finally, we conduct large-scale experiments on UHD VQA databases and the results reveal that, compared to other state-of-the-art VQA methods, our method achieves competitive quality prediction performance with a shorter runtime and fewer model parameters.

## 1. Introduction

UHD video can bring viewers a better visual experience because of its high resolution, high frame rate, high dynamic range and wide color gamut. However, the quality of UHD video is subject to various distortions and noises during the processes of signal capturing, encoding, storing, transmission and displaying. To better monitor and control the video quality in each stage, it is essential to develop an accurate and efficient UHD video-quality-assessment algorithm.

Video quality assessment (VQA) is a technology that can automatically measure video quality. Based on whether the reference video is accessible, VQA can be categorized into three types: full-reference VQA (FR-VQA) [1,2], reduced reference VQA (RR-VQA), and no-reference VQA (NR-VQA). The FR-VQA methods predict the video quality by comparing the reference and distorted videos. The RR-VQA methods employ only partial information from the reference videos for quality prediction. In contrast, the NR-VQA methods can perform quality prediction without using any information from the reference video.

NR-VQA is also known as blind VQA [3,4]. Since no reference video is needed, BVQA can better meet the needs of real-world applications, such as the video quality of experience (QoE). The ITU-T study group designed a no-reference model for UHD videos and standardized it to P.1204.5 [5]. This method uses distorted video and other information, such as the encoding format and bit rate, for quality assessment. Inspired by this method, STEVE et al. proposed the Nofu model [6], which extracts 64 features, such as colorfulness, tone, and saturation from the distorted UHD video, without additional information, and predicts the quality by the support vector regression (SVR). Moreover, most of the current NR-VQA methods are still aimed at low-resolution videos, such as [7,8]. They do not perform well when applied to UHD videos. The main reasons are as follows:

First, the VQA task is closely correlated with the perception of distortion information. However, the current BVQA methods [9,10,11] usually use backbone convolutional neural networks to extract global spatial distortion features. Such networks are designed for computer vision tasks, such as image classification, focusing more on objects rather than distortions. While these methods perform well for those videos with relatively low resolution, because viewers tend to pay more attention to objects which usually appear in the center of the screen, they do not work well for UHD videos. For UHD videos, the attention of viewers is not only focused on the objects in the screen center, but is also attracted by the content in other regions of the screen. These methods that focus on objects cannot fully extract the distortion information outside the object region and, thus, cannot effectively predict the quality of UHD videos. In addition, due to the high resolution of UHD videos, the current methods often need to divide a UHD video frame into image patches of small size, and then perform quality prediction based on image patches instead of the whole video frame. This makes it hard for the backbone neural network to fully extract the global spatial distortion features.

**Figure 1:**
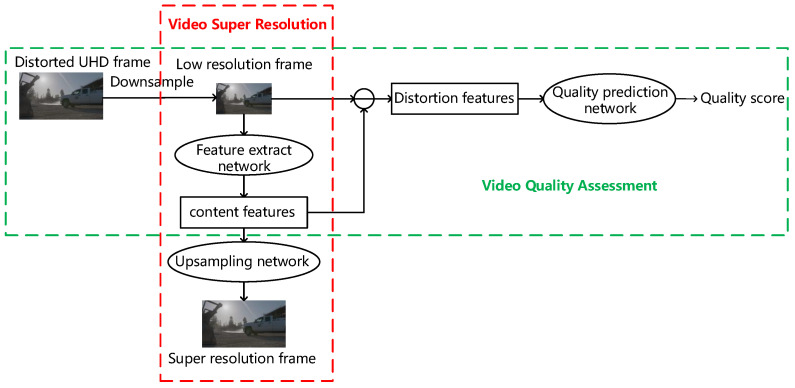
The relationship between video super-resolution and quality assessment. The red box shows the super-resolution process and the green box represents the structure of our method.

In contrast, the video super-resolution task is more related to the VQA task and both of them aim at how to better extract the features related to video quality. The process of video super-resolution involves dealing with the content feature and distortion feature. The content feature is extracted and used to generate high-quality and high-resolution videos, while the distortion information is not used in the super-resolution task. Because the super-resolution model can convert low-resolution video into high-resolution video, the distortion information hidden in the low-resolution video can reflect the distortion distribution of the high-resolution video well. Therefore, we can use the distortion information hidden in the low-resolution video to predict the UHD video quality by embedding the super-resolution model into the VQA model. Figure 1 shows the relationship between the quality assessment and the super-resolution task. The red box in Figure 1 shows the structure of a common super-resolution model. The model first extracts the content features through the super-resolution feature extraction network, and then the content features are used to output high-quality and high-resolution frames by an up-sampling network. The green box in Figure 1 shows the structure of a VQA model proposed in this paper in which the distortion features from the super-resolution model are used in the follow-up quality prediction. Furthermore, in this structure, the input to the feature extraction network is the low-resolution version of the UHD video frame rather than the segmented image patches, so the frame-level global spatial distortion features can be effectively extracted.

**Figure 2:**
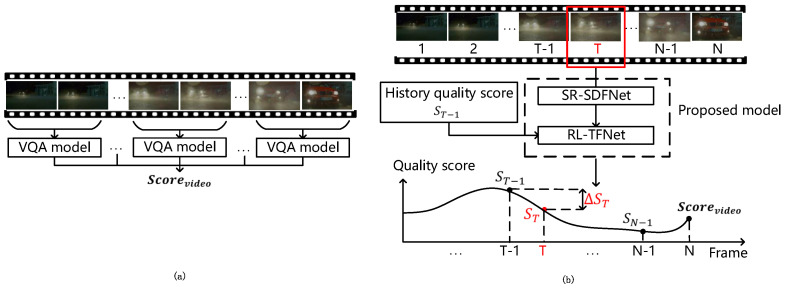
(**a**) Video quality score prediction process of previous NR−VQA methods. (**b**) Video quality score prediction process of our model.

Second, due to the limitation of computing power, most VQA models are unable to continuously handle the temporal features of UHD videos with high frame rates. Instead, these models usually have to segment the video into multiple segments, predict the quality score of each segment, and then average these segment quality scores to obtain the video quality score, as shown in Figure 2a. This kind of operation breaks the temporal correlation of the video sequences and leads to discontinuities in the temporal features. For example, if the quality of the first segment is degraded, it will not affect the quality prediction of the subsequent segment. This is not consistent with the characteristics of human visual perception. Human visual perception of video quality is continuous rather than segment-by-segment, as shown in Figure 2b. To simulate this process, we use a reinforcement learning model to continuously extract the temporal features.

According to the above analysis, we propose a blind video quality assessment method for UHD video based on super-resolution and deep reinforcement learning. First, we construct a spatial distortion feature network based on a super-resolution model (SR-SDFNet) to extract the global spatial distortion features of UHD frames. Then, a time fusion network based on reinforcement learning (RL-TFNet) is employed to aggregate the spatial distortion features of each UHD frame to obtain the UHD video quality score. For the SR-SDFNet, as shown in the green box in Figure 1, the UHD video frames are downsampled before being fed into the super-resolution model for extraction of the global spatial distortion features. This significantly reduces the computational time. For the RL-TFNet, as shown in Figure 2b, the video quality score is adjusted iteratively by combining the global spatial distortion features of each frame with the history quality score. When the last frame is input, the model outputs the final video quality score.

We make three main contributions:

(1) We propose a global spatial distortion feature network based on a super-resolution model (SR-SDFNet) to efficiently and effectively extract the global spatial distortion features from the UHD frames.

(2) We propose a time fusion network based on reinforcement learning (RL-TFNet) to continuously fuse the global spatial distortion features of each frame to obtain the whole video quality score, which can ensure the continuity of the video temporal domain and improve the prediction accuracy for the UHD video quality.

(3) By combining the SR-SDFNet with the RL-TFNet, we present an NR-VQA model BVQA-SR&DRL for UHD videos. Our model is driven by distorted UHD videos without human subjective scores. We evaluate our model on two public UHD-VQA databases. The result demonstrates that our model achieves state-of-the-art performance compared with existing BIQA methods.

## 2. Related Work

### 2.1. BVQA

The current UHD video quality assessment methods usually use the VQA model designed for low-resolution video, apart from the above two methods P.1204.5 [5] and Nofu [6]. Most of these methods are based on machine learning. Zhang et al. [12] used a 3D-DCT transform to extract spatiotemporal features of videos and fed the extracted features into a convolutional neural network to regress perceptual quality scores. CNN-MR [13] and COME [14] used the 2D CNN AlexNet to extract spatial features of videos, which were combined with motion features, and then used support vector regression (SVR) to obtain the final quality score. Chen et al. proposed a concept of temporal resolution and designed RIRNet [15] to extract temporal features at different temporal resolutions for quality perception. Since RIRNet has many temporal resolution inputs, the computational complexity will be higher than for other models. Dendi et al. [16] proposed an asymmetric generalized Gaussian distribution (AGGD) to simulate the MSCN coefficients of distorted video and the statistics of the bandpass filtered output, which are used to predict the quality score by SVR. ChipQA [17] tracked and cropped video regions where motion information existed to obtain localized spatiotemporal slices, and outputted natural video statistical parameters for those extracted slices to perceive video quality. Chen et al. [18] extracted multi-scale distortion features using a VGG16 network combined with an attention mechanism and proposed a pyramid aggregation model along the temporal dimension to obtain the final video-level quality score. Xu et al. [19] extracted time-space features of UGC videos through graph convolution and attention blocks, and built a long short-term memory network to integrate distortion features and obtain the video quality score. Varga et al. [20] proposed an FLG-VQA model which extracts and integrates local and global image statistics features for quality perception. Li et al. [21] proposed a bidirectional GRU network to predict UGC frame scores and integrated the quality scores of multiple frames by constructing temporal memory blocks. According to the characteristics of compressed videos, Lin et al. [22] combined perceivable encoding artifacts (PEAs) detection and visual saliency perception to output the final quality score. However, for UHD videos with large spatial resolution and a high frame rate, the above methods have unsatisfactory performance and slow operation speed. Therefore, our method aims to achieve improvements in terms of both efficiency and effectiveness.

### 2.2. Deep Reinforcement Learning

DRL [23] imitates the learning process of the human brain. The model can find the optimal strategy through constant trials and errors in the environment. In addition, DRL can complete the construction of knowledge models even when environmental information is insufficient. Early works on DRL mainly focused on robotic control and game agents. In recent years, DRL has become a research hotspot in computer vision.

Zhang et al. [24] proposed a DRL model called EBSNet to automatically select appropriate exposure images and combine it with MEFNet to generate high-dynamic-range images. Sun et al. [25] achieved referring expression grounding through a reinforcement learning model, which predicts the possible orientation of the object at each iteration to localize the target object. Nauata et al. [26] combined the DRL network with the GAN network to achieve automated floorplan generation. Wang et al. [27] applied the DRL model, which reduces the computational complexity while ensuring performance, to the field of face video segmentation. Lu et al. [28] regarded coronary CT angiography vessel-level image quality assessment (CCTA VIQA) as a multi-instance learning (MIL) problem, and constructed a reinforcement learning model to gradually select key instances for subsequent quality assessment. Saeed et al. [29] proposed a meta-RL-based meta-learning model to improve the adaptability of the common quality assessment model to the task-specific IQA, such as pneumonia detection in X-ray images and other clinical applications.

However, to our knowledge, RL has never been used for video quality assessment tasks. Existing VQA methods usually divide the entire video into multiple video segments and then process them. We argue that such segmentation is inconsistent with the actual subjective perception process. Therefore, our method attempts to process the entire video with an RL model, which predicts the quality fluctuations that each video frame brings to the entire video, thereby preserving complete and continuous video temporal features.

## 3. Proposed Method

### 3.1. BVQA-SR&DRL

**Figure 3:**
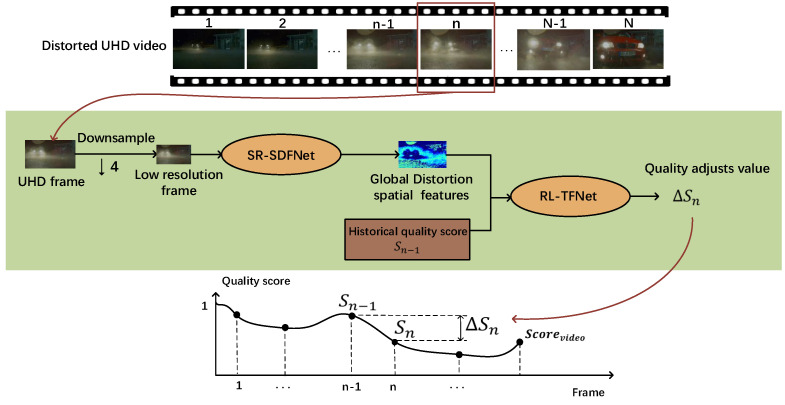
Processing pipeline of the proposed model BVQA−SR&DRL. The SR−SDFNet model is used to extract spatial distortion features from the UHD video frame. Then, the RL-TFNet model observes the spatial features and the historical quality score $S_{n-1}$, which outputs $\Delta S_{n}$ to adjust the quality score. The prediction process of the entire video score is shown in the line graph.

We propose a BVQA method BVQA-SR&DRL that aims to efficiently and accurately predict the quality score of the UHD distorted video. It consists of two components: a spatial distortion feature network based on a super-resolution model (SR-SDFNet) and a time fusion network based on reinforcement learning (RL-TFNet). Figure 3 illustrates the main steps of our method. First, we downsample the UHD video frames to obtain the low-resolution video frames and input them into the SR-SDFNet for spatial distortion feature extraction. Guided by the super-resolution network, the spatial distortion features extracted by the SR-SDFNet can reflect the distortion distribution of the UHD frames well.

Second, the proposed RL-TFNet observes the spatial features extracted by SR-SDFNet, as well as the historical quality score $S_{n-1}$, and outputs the adjust value $\Delta S_{n}$ to increase or decrease the quality score from the first frame, and iteratively adjusts the quality score until the last frame, finally outputting the overall video quality score. The prediction process of the entire video score is shown in the line graph of Figure 3.

### 3.2. Spatial Distortion Feature Network Based on a Super-Resolution Model (SR-SDFNet)

**Figure 4:**
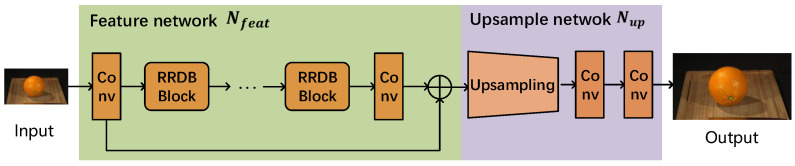
The network structure of the generator network SRResNet.

To extract high-resolution spatial distortion features quickly and completely, we employ the generator network SRResNet used in the super-resolution method ESRGAN [30] to construct SR-SDFNet. Specifically, SRResNet can be divided into a feature network $N_{feat}$ and an upsample network $N_{up}$, as Figure 4 shows. The feature network $N_{feat}$ consists of four residual-in-residual dense blocks (RRDB), which are used to extract content features and distortion features. Each RRDB contains three residual networks and uses dense connections to combine.

In the task of SR, the content features $F\_content_{n}^{L\to H}$ are extracted by the feature network $N_{feat}$ and used to output high-quality frames $I_{n}^{output}$ by an upsample network $N_{up}$, as shown in Formulas (1) and (2). Considering the spatial features of a frame consists of content features and distortion features, we extract the distortion features by making a difference between the frame and the content features in the task of VQA.

(1) $$F\_content_{n}^{L\to H}=N_{\mathrm{feat}\;}\left(I_{n}^{L}\right)$$

(2) $$I_{n}^{output}=N_{\mathrm{up}\;}\left(F\_content_{n}^{L\to H}\right)$$

**Figure 5:**
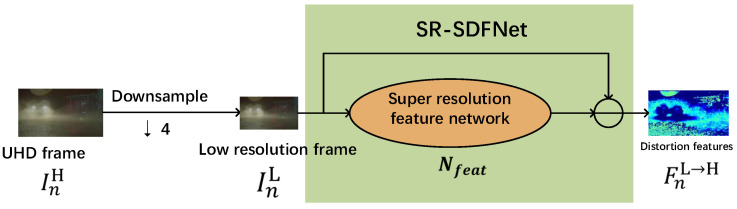
The network structure of the spatial distortion feature network (SR-SDFNet).

In detail, we first downsample the UHD video frame $I_{n}^{H}$ (3840 × 2160) by four times to get the low-resolution video frame $I_{n}^{L}$ (960 × 540). Then, $I_{n}^{L}$ is fed into SR-SDFNET to extract the global spatial distortion features. In detail, SR-SDFNet diffs the $I_{n}^{L}$ with the output of $N_{feat}$ to get the information $F_{n}^{L\to H}$, which is adopted as the distortion feature for the UHD frame, as shown in the Figure 5.

(3) $$I_{n}^{L}=\;\mathrm{Downsampling}\;\left(I_{n}^{H}\right)$$

(4) $$F_{n}^{L\to H}=I_{n}^{L}-N_{\mathrm{feat}\;}\left(I_{n}^{L}\right)$$

To train SR-SDFNET, we first train the super-resolution model ESRGAN based on the VQA database. The inputs to ESRGAN are the distorted frames which are downsampled by a factor of four, and the corresponding reference frames are used as the ground truth of the ESRGAN model. After the losses of ESRGAN stop falling, we use the feature network $N_{feat}$ in SRResNet as the pre-training parameter of SR-SDFNet. Most existing VQA methods use sliding window segmentation on UHD video frames to predict the quality score, which consumes a lot of time and computation. In contrast, the proposed SR-SDFNet only needs to input low-resolution video frames and, thus, most computation is performed in the low-resolution feature space. In this way, the algorithm efficiency is greatly improved. Furthermore, because the spatial distortion feature extraction is based on the video frames instead of the segmented image patches, the integrity of the spatial distortion feature is guaranteed.

### 3.3. Time Fusion Network Based on Reinforcement Learning (RL-TFNet)

**Figure 6:**
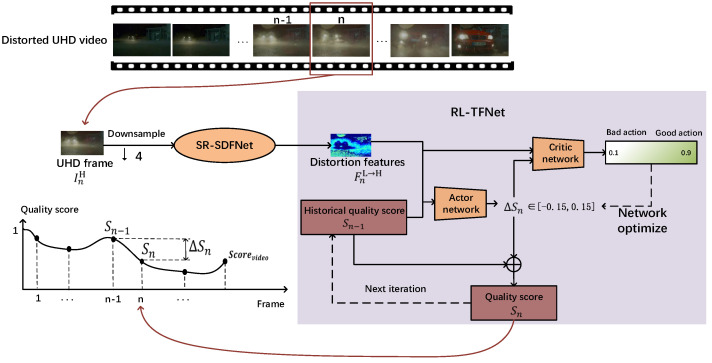
The processing pipeline of the time fusion network based on reinforcement learning (RL-TFNet).

To continuously integrate the spatial quality features extracted by SR-SDFNet in the temporal dimension, we formulate the video quality assessment task as a Markov decision process (MDP) and propose the RL-TFNet model, which simulates the process of human visual perception of video quality. As shown in Figure 6, we initially set the video quality score $S_{0}$ to one (i.e., the highest quality). The RL-TFNet model consists of the actor network and critic network, in which the actor network iteratively adjusts the quality score according to the spatial distortion features extracted and the historical quality score, as shown in the formula. In addition, the critic network outputs action values to optimize the quality perception of the actor network.

Since the subjective quality scores of each video frame used as the ground truth are hard to obtain, the training of RL-TFNet is guided by a reward generated from VAMF-4K [31], which is a widely used FR-VQA method based on VMAF. VMAF-4K mainly extracts three features, the visual quality fidelity (VIF) [32], a detail loss measure (DLM), and temporal information (TI), and integrates them to calculate the video quality score. The proposed RL-TFNet contains the following important factors: the state space, the action space and the definition of the reward function, which will be introduced in detail.

#### 3.3.1. State Space

In order for our model to continuously fuse long-range UHD video quality features, we define the state vector as $\;\mathrm{state}{\;}_{n}=\left\{F_{n}^{L\to H},S_{n-1}\right\}$, where $F_{n}^{L\to H}$ represents the spatial distortion feature extracted by SR-SDFNet for the n-th frame. $S_{n-1}$ means the historical quality score from the first frame to the n-1th frame, which ranges from 0 to 1, and the larger the value, the higher the video quality. The initial preset historical quality score $S_{0}$ is 1. The model integrates the spatial distortion features of the nth frame with the historical quality of the previous n-1 frames by observing $F_{n}^{L\to H}$ and $S_{n-1}$.

#### 3.3.2. Action Space

For the n-th video frame $I_{n}^{H}$, RL-TFNet outputs action $a_{n}$ by observing $state_{n}$ to adaptively adjust the historical quality score $S_{n-1}$, where action $a_{n}$ is a continuous value taken from [−0.15, 0.15]. When the quality of the nth frame is improved compared to the previous n-1 frames, and $a_{n}$ is a positive value, the quality score $S_{n}$ becomes higher than $S_{n-1}$. Conversely, when the quality of the nth frame is lower than the previous n − 1 frames, the output $a_{n}$ is between [−0.15, 0], the quality score $S_{n}$ becomes lower. Our model adjusts the historical quality score $S_{n-1}$ through action $a_{n-1}$, so that the quality score $S_{n}$ is closer to the subjective perception of the previous n frame.

#### 3.3.3. Reward Function

The reward is an essential metric for the agent to learn which action performs better in the environment. Considering the frame $I_{n}^{H}$ and the current quality score $S_{n-1}$, the reward should encourage the model to increase or decrease the video quality score. The reward function in our method is shown in the formula.

(5) $$r_{n}=-\left|a_{n}-\Delta S_{n}^{vmaf}\right|$$

(6) $$\Delta S_{n}^{vmaf}=S_{n}^{vmaf}-\frac{\sum_{i=1}^{n-1}S_{i}^{vmaf}}{n-1}$$

$S_{n}^{vmaf}$ represents the quality score of the nth frame calculated by the full reference model VMAF-4K. The higher the score, the higher the quality. $\frac{\sum_{i=1}^{n-1}S_{i}^{vmaf}}{n-1}$ represents the average VMAF-4K score of the 1st to n−1th video frames, and $\Delta S_{n}^{vmaf}$ represents the quality fluctuation of in the nth frame. When $a_{n}$ is closer to $\Delta S_{n}^{vmaf}$, $r_{n}$ will be larger, otherwise, $r_{n}$ will be smaller.

#### 3.3.4. Network Introduction

**Figure 7:**
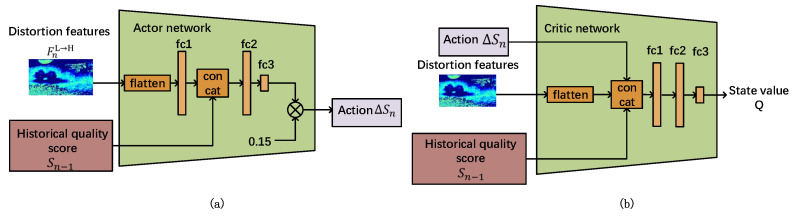
(**a**) The network structure of the actor network. (**b**) The network structure of the critic network.

After defining the above components of MDP, we design and update RL-TFNet via a deep deterministic policy gradient (DDPG) [33]. RL-TFNet consists of an actor network and a critic network, as shown in Figure 7. The input of the actor network is the state vector $state_{n}=\left\{F_{n}^{L\to H},S_{n-1}\right\}$, and the corresponding output is the adjusted value $\Delta S_{n}$ to the quality score. The critic network criticizes the action $\Delta S_{n}$ based on the state $state_{n}$ and outputs the state value Q. Both networks have a copy network for calculating the target value—the structure is the same as the main network, but the parameter update speed is different. In each training iteration, both the actor and critic networks are optimized sequentially, as shown in Algorithm 1. In the inference stage, only the actor network is used to predict actions to adjust the quality score.

**Algorithm 1** RL-TFNet AlgorithmRandomly set the initialize weights of the actor network $\pi \left(State|\theta^{\pi }\right)$ and critic network $Q\left(State,A|\theta^{Q}\right)$. Initialize copy network weights $\theta^{Q^{\prime }}\leftarrow \theta^{Q}$, $\theta^{\pi^{\prime }}\leftarrow \theta^{\pi }$. Initial quality score $S_{0}=1$
1:**for**trainstepn∈[1,N]**do**2: Set $state_{n}=\left\{F_{n}^{L\to H},S_{n}\right\}$;3: Select action $a_{n}=\pi \left(state_{n}|\theta^{\pi }\right)+Noise$;4: Execute action on quality score $S_{n+1}=S_{n}+a_{n}$ and get reward $r_{n}$;5: Set $state_{n+1}=\left\{F_{n+1}^{L\to H},S_{n+1}\right\}$;6: Store transition $\left(state_{n},a_{n},r_{n},state_{n+1}\right)$ in replay buffer;7: Set $y_{n}=\gamma Q^{\prime }\left(state_{n+1},\pi^{\prime }\left(state_{n+1}|\theta^{\pi^{\prime }}\right)|\theta^{Q^{\prime }}\right)$$+r_{n}$8: Minimize the loss to optimize critic network: $L_{critic}=\frac{1}{m}\sum_{n}{\left(y_{n}-Q\left(state_{n},a_{n}|\theta^{Q}\right)\right)}^{2}$9: Optimize actor network by the cost function: $J\left(\theta^{\pi }\right)=\frac{1}{m}\sum_{n}Q\left(state_{n},a_{n}|\theta^{Q}\right)$10: Update the copy network weights: $\theta^{Q^{\prime }}\leftarrow \tau \theta^{Q}+\left(1-\tau \right)\theta^{Q^{\prime }}$ $\theta^{\pi^{\prime }}\leftarrow \tau \theta^{\pi }+\left(1-\tau \right)\theta^{\pi^{\prime }}$11:**end for**


### 3.4. Training Procedure

In our method, we first extract global spatial distortion features $F_{n}^{L\to H}$ from the n-th UHD distorted frame $I_{n}^{H}$ through SR-SDFNet, and the extracted features $F_{n}^{L\to H}$ and historical quality score $S_{n-1}$ are used as the input of RL-TFNet to output $\Delta S_{n}$ which adjusts the historical quality score $S_{n-1}$ and get the current quality score $S_{n}$. The above process continues from the first frame to the last frame to get the entire video quality score, as shown in the formula.

(7) $$F_{n}^{L\to H}=N_{\mathrm{SR}-\mathrm{SDFNet}}\left(\;\mathrm{downsampling}\;\left(I_{n}^{H}\right)\right)$$

(8) $$\Delta S_{n}=N_{RL-VQANet}\left(F_{n}^{L\to H},S_{n-1}\right)$$

(9) $$S_{n}=S_{n-1}+\Delta S_{n}$$

To train the two networks (SR-SDFNet and RL-TFNet) stably, we first train the super-resolution model ESRGAN, the distorted frames are downsampled by a factor of four as the input of ESRGAN, and the corresponding reference frames are used as the ground truth of the super-resolution model. After the ESRGAN is trained to convergence, we use the feature network $N_{feat}$ in the ESRGAN generator network SRResNet as the pre-training parameters of SR-SDFNet, and then we jointly train the two networks (SR-SDFNet and RL-TFNet).

## 4. Experimental Results

### 4.1. Databases and Evaluation Criteria

To train the proposed model, we collect 50 UHD (3840 × 2160 pixels and 50 fps) video sequences from the source materials of the UHD TV programs. These source materials are only slightly compressed, so the video quality is high enough to be used as reference videos. The collected video sequences cover a wide range of content, including outdoor, indoor, night scenery, buildings, people, sports games and so on. The duration of each sequence is 10 s to 15 s. We manually add three types of compression distortion (AVC, HEVC and VP9) to each of these UHD sequences;each type has four levels, which are set according to the MCML [34] database. Finally, our training database contains 50 reference videos and 600 distorted videos.

In order to evaluate the performance of our method, two UHD VQA databases are used for testing: the AVT-VQDB-UHD-1 [35] and the MCML [34] database. The AVT-VQDB-UHD-1 database includes 13 open-source reference videos and 432 distorted videos, which correspond to four different subjective tests. TEST_1 contains three different codecs (H.264, H.265 and VP9) and four resolutions from 360 p to 2160 p, TEST_2 considers two codecs (H.264 and H.265) and four different bits-per-pixel (bpp) values. TEST_3 uses the same bpp values as TEST_2 and encodes videos with H.265 and VP9 codecs. Test videos for TEST_4 include four frame rates from 15 fps to 60 fps and six different resolutions, which are encoded with H.264. Each distorted video has a mean opinion score (MOS), which ranges from 1 to 5. The MCML database consists of 10 reference videos and 240 distorted videos, which have two resolutions (1920 × 1080 and 3840 × 2160) and three compression distortions (AVC, HEVC and VP9) with four different levels.

We chose Spearman’s rank order correlation coefficient (SROCC), the linear correlation coefficient (PLCC) and root-mean-square error (RMSE) to measure the VQA model performance. SROCC describes the rank consistency of the predicted score and the subjective score. Where N is the total number of distorted videos, and $d_{n}$ represents the ranking difference between the subjective score and the predicted score of the n-th video.

(10) $$\;\mathrm{SROCC}\;=1-\frac{6\sum_{n}d_{n}^{2}}{N\left(N^{2}-1\right)}$$

PLCC describes the linear correlation between the predicted score and the subjective score, where $s_{i}$ is the predicted quality score and $p_{i}$ is the video subjective score. $\bar{s}$ and $\bar{p}$ denote the average of the predicted scores and the average of the subjective scores, respectively.

(11) $$\mathrm{PLCC}=\frac{\sum_{i=1}^{N}\left(s_{i}-\bar{s}\right)\left(p_{i}-\bar{p}\right)}{\sqrt{\sum_{i=1}^{N}{\left(s_{i}-\bar{s}\right)}^{2}\sum_{i=1}^{N}{\left(p_{i}-\bar{p}\right)}^{2}}}$$

RMSE is mainly used to measure the relative error, where $s_{p}$ is the predicted quality score and $s_{m}$ is the video subjective score.

(12) $$\mathrm{RMSE}=\sqrt{\frac{\sum {\left(S_{p}-S_{m}\right)}^{2}}{n-1}}$$

Following Zhang et al. [36] and Ma et al. [37], we estimate the parameters of a nonlinear function that transforms the prediction values to the same scales as the subjective score.

(13) $$\tilde{q}=\left(\eta_{1}-\eta_{2}\right)/\left(1+exp\left(-\left(\hat{q}-\eta_{3}\right)/\left|\eta_{4}\right|\right)\right)+\eta_{2}$$

### 4.2. Implementation Details

In the training session, the SR model ESRGAN is first trained on the training database according to [30]. The distorted frames in our training database are downsampled by a factor of four as the input of ESRGAN. Then the SR-SDFNET and RL-TFNet are jointly trained in the training database. The video frames used for training are obtained by sampling one frame every 30 frames of each video in the training database. The Adam algorithm is used to optimize the model. The learning rate is set to 1 × 10^−4^ and decays by a factor of 0.5 every 10,000 iterations. We choose the model with the largest reward value as the best model. In the testing session, each test video is frame sampled every 30 frames intervals for score prediction.

### 4.3. Comparisons with the State of the Art

In this section, we evaluate the performances of our model and various other BVQA models on two UHD-VQA databases. The main models being compared have all been designed for video quality assessment in recent years, and include VSFA [38], TLVQM [39], VIDEVAL [40], GSTVQA [18], RAPIQUE [10], ChipQA [17], NOFU [6], HEKE [9] and HFR-BVQA [11]. Among them, VIDEVAL, ChipQA, TLVQM, RAPIQUE, and VSFA are five VQA models for UGC video. To ensure fairness of comparison, we train the nine BVQA models on the KONVID [41] database and test them on the MCML and AVT-VQDB-UHD-1 databases. Such cross-dataset experiments can also give the generalization performance of these models. The overall testing results are shown in Table 1 and Table 2. The best results are highlighted in bold.

It can be observed from Table 1 and Table 2 that our model obtains superior performance compared to other BVQA algorithms on both the two databases. Compared with previous methods, our model can extract the distortion features of large-resolution videos more completely. Moreover, the above results illustrate the strong generalizability of our model on UHD distortion databases. This is because the previous methods are supervised by MOS, whereas our model is driven by the super-resolution process without human subjective scores and can adapt to different UHD video quality databases.

To our surprise, the proposed model is weaker than HFR-BVQA for TEST_4 on the AVT-VQDB-UHD-1 database. One reason for this is that the TEST_4 of AVT mainly contains distorted videos with different frame rates. Compared with our method, HFR-BVQA is specially designed for low-frame-rate videos. In response to this phenomenon, we will make special optimizations for distorted videos with different frame rates in the future.

### 4.4. Performance on Computation Complexity and Runtime

**Figure 8:**
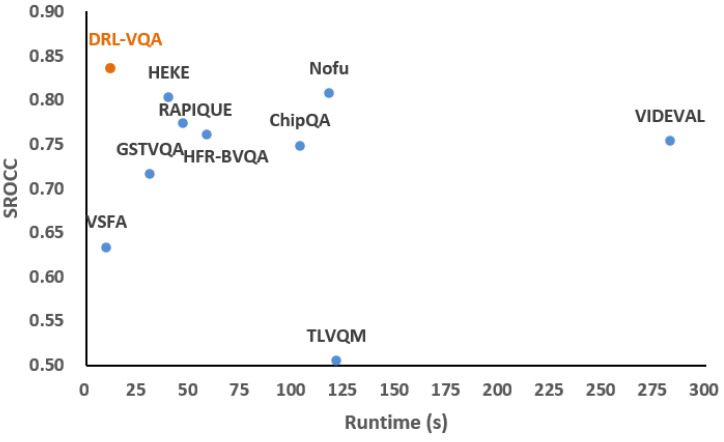
The speed and performance comparison for our model and the other nine BVQA models on the MCML database.

To evaluate the time and spatial complexity of the proposed model, our model is compared with the other nine BVQA models: VSFA, TLVQM, VIDEVAL, GSTVQA, RAPIQUE, ChipQA, NOFU, HEKE and HFR-BVQA. These models are implemented on CPU or GPU, as requested by the authors. The hardware platform used for testing includes an NVIDIA TITAN X GPU processor and Core i7-5930K CPU @ 3.5 GHz. We summarize the runtime and network parameter size in Table 3. The runtime represents the average time to predict a UHD video with a resolution of 3840 × 2160 and 300 frames from the MCML database. In addition, Figure 8 is a scatterplot of speed and performance comparison for our model and the other nine BVQA models in MCML to visually compare.

As shown in Table 3, our model is faster than most BVQA models and the parameter size is smaller than those of the above methods. It should be noted that the previous methods take more time to extract the spatial features of large-resolution video frames. In contrast, our method downsamples the video frames from 3840 × 2160 to 960 × 540 for processing, which greatly improves the running speed. In addition, some models extract spatial features based on basic CNN models, such as GSTVQA using the VGG16 model, and RAPIQUE and HFR-BVQA using the Resnet-50 model. In contrast, our method employs a lightweight super-resolution model for feature extraction, which can predict video quality with smaller model parameters. Furthermore, although the spatial and time complexity of VSFA is slightly lower than that of our model, our model has a comprehensive advantage in both performance and complexity, as shown in Figure 8.

### 4.5. Visual Analysis

**Figure 9:**
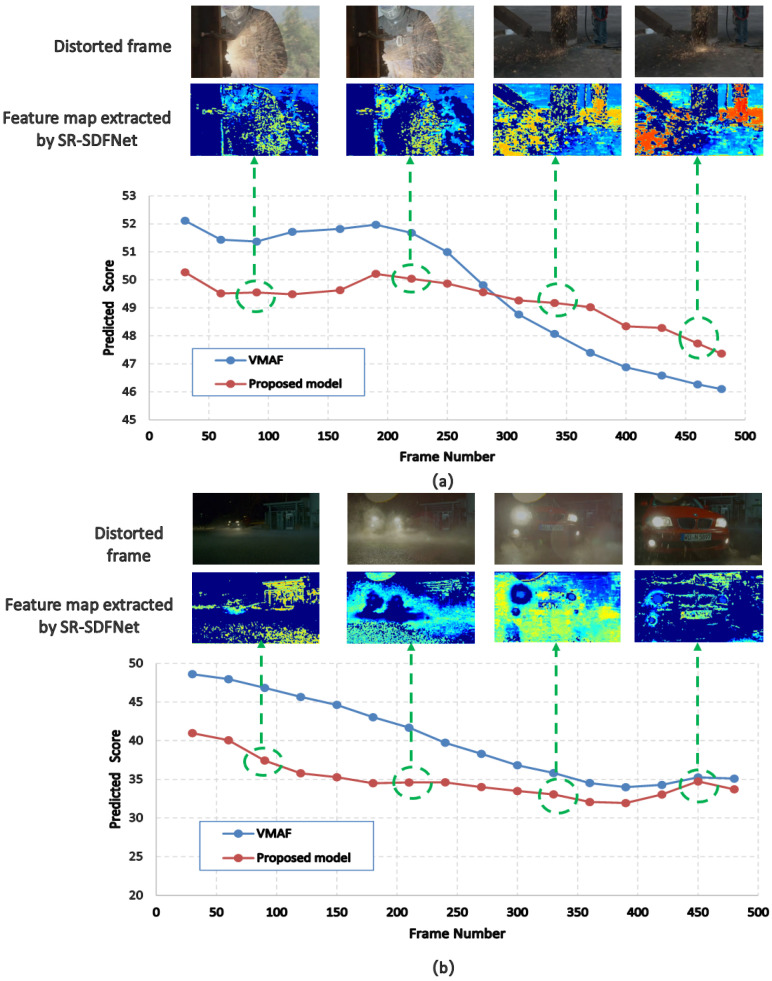
Two visualization examples about the prediction quality score process of our model. (**a**) and (**b**) respectively represent two distorted videos from the AVT-VQDB-UHD-1 database and curves of their predicted quality scores.

In order to visually verify the effectiveness of our model, we output the feature map from the spatial distortion feature network (SR-SDFNet) and show the fluctuation of video quality over time through a line graph, as shown in Figure 9, where the red dot is the quality scores calculated by the FR-VQA method VMAF-4K and the blue dots are the quality scores predicted by the proposed method. Here, we use the VMAF-4K method to reflect the real quality distribution of the distorted video. As shown in Figure 9a, the trends in the predicted quality scores in our model are similar to those of VMAF-4K, which is gradually degraded. The feature map shows that, along with the degradation of the quality scores, the distortion features are increasing. The same phenomenon can also be observed in Figure 9b. We find that the predicted scores of the proposed model are consistent with VMAF-4K. As time changes, the video quality is first degraded and then improved. Similarly, the spatial distortion features in the feature map are also increased and then reduced. This suggests that our model can perceive the distortion features and accurately predict the video quality.

### 4.6. Ablation Study

In this section, we investigate the functionalities of SR-SDFNet and RL-TFNet. All experiments are evaluated on the AVT-VQDB-UHD-1 and MCML databases.

#### 4.6.1. SR-SDFNet

To verify the effectiveness of the proposed SR-SDFNet, we evaluate the performance of different spatial feature extractors. The analysis includes five kinds of networks, Resnet-50 [42], VGG16 [41], Mobilenetv2 [43], RCAN [44] and SR-SDFNet (proposed). RCAN is a super-resolution network, which is implemented by the residual-in-residual (RIR) structure and the channel attention mechanism. Table 4 shows the PLCC, SROCC and network parameter size on two databases. We can observe from the results that SR-SDFNet is superior to the other four spatial feature extractors. This is because the three extractors (Resnet-50, VGG16 and Mobilenetv2) are designed for other computer vision tasks, such as image classification. In contrast, SR-SDFNet and RCAN are built based on a video quality enhancement model, which is more suitable for the VQA task. Moreover, the RCAN network only takes a single LI loss as a loss function, and SR-SDFNet introduces perceptual loss and GAN network loss through the pre-training of ESRGAN, so that SR-SDFNet can better extract spatial distortion features with the smallest parameters than other networks.

**Figure 10:**
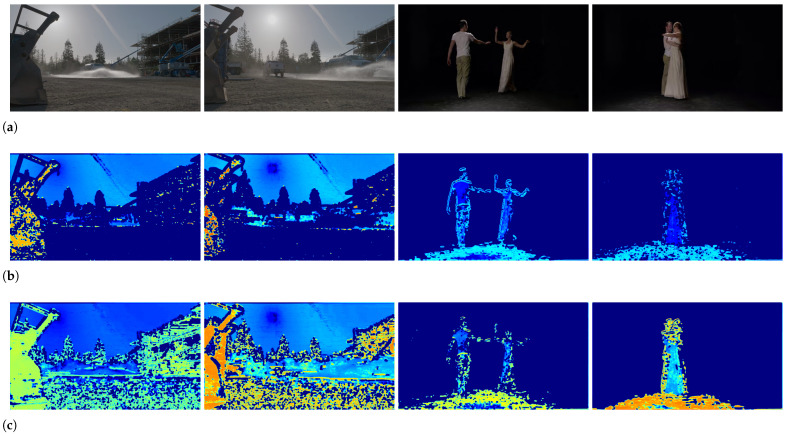
Visual comparison of spatial feature extraction. (**a**) shows the distorted UHD frames. (**b**) presents the feature maps produced by the Resnet-50 model. (**c**) presents the feature maps produced by the proposed SR-SDFNet model.

Furthermore, Figure 10 shows some visual examples of feature maps from SR-SDFNet and Resnet-50. The results in Figure 10c are produced by the SR-SDFNet model and the results in Figure 10b are produced by the Resnet-50 model. We can intuitively find that the Resnet-50 model mainly extracts the local edge of the object, and SR-SDFNet pays more attention to the distortion area of the frames. It can be concluded that the proposed SR-SDFNet is suitable for the UHD-VQA task.

#### 4.6.2. RL-TFNet

We use two time-process strategies to explore the effectiveness of the proposed RL-TFNet. In the first strategy, we first extract spatial features through SR-SDFNet and then regress the features of each frame to a quality score through two fully connected layers. In testing, we average the prediction scores for multiple frames as the score of the entire video. In the second strategy, the spatial features from multiple frames are fed into an LSTM network [45] to predict quality scores. The comparison results are shown in Table 5 and demonstrate that the RL-TFNet performs better than the other two strategies. This occurs because the average pooling in the first strategy only deals with spatial features and the LSTM model in the second strategy has difficulty fusing long-range temporal features.

## 5. Conclusions

In this paper, we present a fast and accurate blind quality assessment algorithm for UHD videos. It consists of two components: a spatial distortion feature network based on a super-resolution model (SR-SDFNet) and a time fusion network based on reinforcement learning (RL-TFNet) in which SR-SDFNet can employ a super-resolution model to efficiently extract the spatial distortion features and RL-TFNet is used to adjust the quality score in the continuous time dimension. Extensive experimental results demonstrate that the proposed model outperforms all compared competing BVQA methods on UHD quality databases. Furthermore, our model has a smaller parameter size and runs faster than other methods, which makes it more suitable for practical applications in UHD VQA.

## Figures and Tables

**Table 1:** Performance comparison on the MCML databases.

Method	ALL			AVC			HEVC			VP9		
	SRCC	PLCC	RMSE	SRCC	PLCC	RMSE	SRCC	PLCC	RMSE	SRCC	PLCC	RMSE
VSFA	0.633	0.708	1.751	0.757	0.834	1.613	0.614	0.655	1.892	0.295	0.575	1.399
TLVQM	0.505	0.577	2.050	0.607	0.629	1.936	0.487	0.526	2.156	0.462	0.449	1.790
VIDEVAL	0.753	0.686	1.808	0.751	0.828	1.625	0.811	0.834	1.384	0.698	0.704	1.244
GSTVQA	0.716	0.830	1.382	0.740	0.855	1.513	0.774	0.850	1.319	0.588	0.682	1.251
RAPIQUE	0.774	0.758	1.617	0.781	0.763	1.887	0.818	0.828	1.402	0.726	0.779	1.074
ChipQA	0.748	0.753	1.636	0.755	0.738	1.821	0.792	0.791	1.565	0.702	0.753	1.068
Nofu	0.808	0.803	1.544	0.832	0.803	1.674	0.838	0.824	1.502	0.726	0.754	1.068
HEKE	0.803	0.834	1.487	0.816	0.811	1.657	0.810	0.845	1.485	0.722	0.755	1.065
HFR-BVQA	0.761	0.775	1.600	0.808	0.780	1.828	0.836	0.847	1.463	0.712	0.696	1.456
**Proposed**	**0.836**	**0.878**	**1.189**	**0.874**	**0.841**	**1.387**	**0.869**	**0.896**	**1.110**	**0.733**	**0.789**	**1.050**

The best results are highlighted in bold.

**Table 2:** Performance comparison on the AVT-VQDB-UHD-1 databases.

Method	ALL			TEST1			TEST2			TEST3			TEST4		
	SRCC	PLCC	RMSE	SRCC	PLCC	RMSE	SRCC	PLCC	RMSE	SRCC	PLCC	RMSE	SRCC	PLCC	RMSE
VSFA	0.607	0.601	0.826	0.715	0.692	0.768	0.754	0.789	0.641	0.696	0.729	0.738	0.536	0.596	0.737
TLVQM	0.488	0.509	0.977	0.501	0.501	0.992	0.510	0.524	0.963	0.516	0.537	0.987	0.469	0.477	0.921
VIDEVAL	0.715	0.724	0.686	0.704	0.722	0.738	0.739	0.756	0.668	0.747	0.757	0.711	0.668	0.679	0.641
GSTVQA	0.679	0.701	0.737	0.536	0.655	0.803	0.777	0.835	0.573	0.780	0.814	0.626	0.688	0.731	0.626
RAPIQUE	0.674	0.729	0.708	0.500	0.696	0.763	0.837	0.866	0.521	0.767	0.797	0.651	0.680	0.757	0.599
ChipQA	0.725	0.745	0.667	0.714	0.761	0.716	0.766	0.718	0.703	0.740	0.778	0.691	0.652	0.689	0.637
Nofu	0.731	0.701	0.774	0.798	0.745	0.709	0.795	0.746	0.741	0.748	0.682	0.823	0.632	0.600	0.803
HEKE	0.762	0.775	0.641	0.780	0.801	0.680	0.805	0.819	0.617	0.760	0.745	0.703	0.684	0.707	0.638
HFR-BVQA	0.711	0.750	0.671	0.750	0.756	0.721	0.717	0.764	0.661	0.709	0.720	0.757	**0.723**	**0.733**	**0.615**
**Proposed**	**0.816**	**0.803**	**0.616**	**0.849**	**0.859**	**0.652**	**0.878**	**0.866**	**0.521**	**0.807**	**0.847**	**0.572**	0.717	0.729	0.628

The best results are highlighted in bold.

**Table 3:** Performance on computation complexity.

Method	Implement	Runtimes (s)	Parameters (Millions)
TLVQM	Matlab-CPU	121.20	-
VIDEVAL	Matlab-CPU	282.46	-
RAPIQUE	Matlab-CPU	46.54	96.4
HFR-BVQA	Matlab-CPU	58.12	97.1
ChipQA	Python-CPU	103.40	-
Nofu	Python-CPU	117.52	-
VSFA	Python-GPU	9.74	100
GSTVQA	Python-GPU	30.88	580.4
HEKE	Python-GPU	39.90	377.6
Proposed	Python-GPU	12.08	6.2

**Table 4:** Performance comparison of different spatial feature extractors on two databases.

Method	MCML		AVT		Parameters
	SRCC	PLCC	SRCC	PLCC	
VGG16	0.795	0.818	0.788	0.772	528 M
Mobilenetv2	0.762	0.786	0.723	0.738	5.2 M
Resnet-50	0.813	0.854	0.793	0.774	97.8 M
RCAN	0.829	0.870	0.811	0.789	59.7 M
SR-SDFNet	0.836	0.878	0.816	0.803	4.9 M

**Table 5:** Performance comparison of different time-process strategies on two databases.

Time Process Strategies	MCML		AVT	
	SRCC	PLCC	SRCC	PLCC
Average Pooling	0.792	0.795	0.767	0.773
LSTM	0.802	0.820	0.781	0.772
RL-TFNet	0.836	0.878	0.816	0.803

## Data Availability

The datasets used were obtained from public, open-source datasets: 1. AVT-VQDB-UHD-1: https://github.com/Telecommunication-Telemedia-Assessment/AVT-VQDB-UHD-1 (accessed on 30 October 2019). 2. MCML: https://mcml.yonsei.ac.kr/downloads/4kuhdvideoquality (accessed on 10 June 2020).
